# Regulation of heterogeneous cancer-associated fibroblasts: the molecular pathology of activated signaling pathways

**DOI:** 10.1186/s13046-020-01611-0

**Published:** 2020-06-16

**Authors:** Go J. Yoshida

**Affiliations:** Department of Immunological Diagnosis, Juntendo University Graduate School of Medicine, 2-1-1, Hongo, Bunkyo-ku, Tokyo, 113-8421 Japan

**Keywords:** Cancer-associated fibroblasts, Canonical Wnt signaling pathway, Collective cell migration, Drug repositioning, Heterogeneity, Interstitial fluid pressure, Receptor tyrosine kinase, Stromal stiffness, TGF-β, YAP/TAZ

## Abstract

Accumulating evidence indicates that intratumoral heterogeneity contributes to the development of resistance to anticancer therapeutics. Fibroblasts, which are components of the paraneoplastic stroma, play a crucial role in the wound-healing process. Activated fibroblasts accumulate in the wound and are involved in many aspects of the tissue remodeling cascade that initiates the repair process and prevents further tissue damage. The pathophysiological roles of cancer-associated fibroblasts (CAFs) in the heterogeneous tumor microenvironment have attracted increasing interest. CAFs play crucial roles in tumor progression and the response to chemotherapy. Several cytokines and chemokines are involved in the conversion of normal fibroblasts into CAFs, and some of these form a feedback loop between cancer cells and CAFs. In addition, the physical force between tumor cells and CAFs promotes cooperative invasion or co-migration of both types of cells. Pro-inflammatory cytokines, such as leukemia inhibitory factor (LIF) and interleukin-6 (IL-6), are secreted by both cancer cells and CAFs, and mediate the epigenetic modification of CAFs. This enhances the pro-tumorigenic function of CAFs mediated by promoting actomyosin contractility and extracellular matrix remodeling to form the tracks used for collective cancer cell migration. The concept of intra-tumoral CAF heterogeneity refers to the presence of inflammatory CAFs with low levels of α-smooth muscle actin (α-SMA) and high levels of IL-6 expression, which are in striking contrast to transforming growth factor-β (TGF-β)-dependent myofibroblastic CAFs with high α-SMA expression levels. CAF populations that suppress tumor growth and progression through stroma-specific Hedgehog (Hh) activation have been detected in different murine tumor models including those of the bladder, colon, and pancreas. A new therapeutic strategy targeting CAFs is the “stromal switch,” in which tumor-promoting CAFs are changed into tumor-retarding CAFs with attenuated stromal stiffness. Several molecular mechanisms that can be exploited to design personalized anticancer therapies targeting CAFs remain to be elucidated. Strategies aimed at targeting the tumor stroma as well as tumor cells themselves have attracted academic attention for their application in precision medicine. This novel review discusses the role of the activation of EGFR, Wnt/β-catenin, Hippo, TGF-β, and JAK/STAT cascades in CAFs in relation to the chemoresistance and invasive/metastatic behavior of cancer cells. For instance, although activated EGFR signaling contributes to collective cell migration in cooperation with CAFs, an activated Hippo pathway is responsible for stromal stiffness resulting in the collapse of neoplastic blood vessels. Therefore, identifying the signaling pathways that are activated under specific conditions is crucial for precision medicine.

## Background

Fibroblasts are spindle-shaped cells that secrete collagen and have a cytoplasm with a predominant rough endoplasmic reticulum. Fibroblasts synthesize the extracellular matrix (ECM) of the connective tissue and play a crucial role in maintaining the structural integrity of most tissues including the skin [1–3]. The mammalian dermis represents an archetypal mesenchymal tissue that is largely composed of ECM elements, including type I and type III collagens, as well as proteoglycans and elastin [3]. Fibroblast heterogeneity depends on developmental stage and the tissue microenvironment [4, 5].

Fibroblasts exhibit distinct cellular phenotypes according to the surrounding microenvironment. Activated fibroblasts in tumor tissues are defined as cancer-associated fibroblasts (CAFs) [6–9]. In recent years, extensive research demonstrated that CAFs are the major cellular components of the tumor microenvironment in both primary and metastatic tumors; CAFs contribute to the regulation of a series of steps critical for malignant progression, including cancer initiation, proliferation, invasion, and metastasis, by producing various types of cytokines, chemokines, growth factors, and matrix-degrading enzymes [6, 8, 9]. CAFs are distinguished from their normal counterparts by the differential expression of markers such as α-smooth muscle actin (α-SMA), fibroblast activation protein (FAP), fibroblast-specific protein 1 (FSP1), and platelet-derived growth factor receptor (PDGFR) [7–9]. In addition to these markers, three proteins including collagen 11-α1, microfibrillar-associated protein 5, and asporin tend to be exclusively expressed in CAFs [9, 10]. The recent identification of proteins whose expression is restricted to CAFs may improve the reliable identification of CAFs and increase their value as candidate biomarkers and therapeutic targets.

**Fig. 1:**
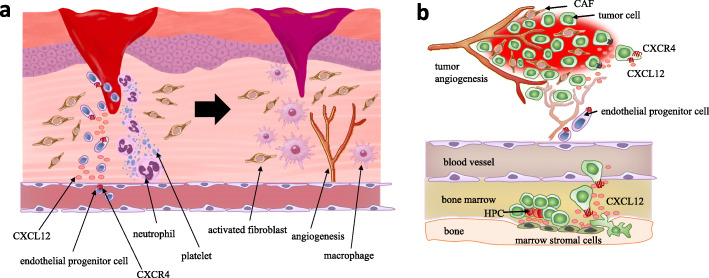
Activated fibroblasts in granulation tissue and carcinoma-associated fibroblasts (CAFs) closely resemble each other regarding their association with the microenvironment. **a** Hemostasis occurs at the first stage of wound healing, followed by the formation of clots by platelets at the injury site, which changes into fibrin (left). Neutrophils migrate to the granulation tissue, and cytokines are secreted, whereas activated fibroblasts are recruited to the wound-healing tissue. Endothelial progenitor cells are recruited in a manner dependent on the CXCL12-CXCR4 axis, which contributes to angiogenesis (right). During the proliferation stage of wound healing, macrophages infiltrate and initiate phagocytosis for the deposition of new extracellular matrix (ECM) [14]. **b** Robust neoangiogenesis occurs in the hypoxic tumor microenvironment. Unlike normal fibroblasts, CAFs stimulate tumor progression by secreting CXCL12. CXCL12 derived from CAFs promotes the recruitment of CXCR4-positive endothelial progenitor cells, which are essential for angiogenesis. Invasive metastasis to the bone marrow with high CXCL12 expression triggers CXCR4 activation in circulating tumor cells, which “hijack” the CXCL12-CXCR4 axis for homing to microenvironments that are normally restricted to hematopoietic progenitor cells (HPCs) [16]

CAFs and activated fibroblasts play similar roles in wound healing and fibrosis [11–14]. Fibroblasts are essential for tissue repair after damage and are involved in wound contraction, deposition of granulation tissue, and production and remodeling of the ECM in parallel with recruitment of platelets, neutrophils, and macrophages [14, 15] (Fig. 1). Fibroblasts in granulation tissue acquire a myofibroblastic phenotype characterized by α-SMA expression. On the other hand, CAFs alter the microenvironment by directly interacting with cancer cells and regulating paracrine signaling via inflammatory cytokines, control the immune response to neoplasia, deposit diverse ECM components, stimulate angiogenesis, and provide a scaffold for tumor metastasis and invasion [8].

CAFs can be recruited to the tumor from a distant source such as the bone marrow [7, 17]. The trans-differentiation of epithelial cells and pericytes can also give rise to CAF-like populations in response to epithelial-mesenchymal transition (EMT) and endothelial-mesenchymal transition (EndoMT), respectively [9, 18, 19]. To define and identify the origin of CAFs, it is important to consider that CAFs are ‘activated fibroblasts’, which, by striking contrast to non-activated (quiescent) tissue-resident fibroblasts, are an expanding population of cells that either proliferates in situ or is recruited to the tumor [7, 20]. The key features of CAFs that distinguish them from quiescent fibroblasts include metabolic adaptations that support their need for enhanced proliferation and biosynthetic activities, such as the production of ECM components and cytokines, growth factors, and enzymes to remodel the stroma [7, 9, 21]. However, the cellular origin of CAFs and the mechanisms underlying the reprogramming of normal fibroblasts into CAFs remain largely unknown.

The heterogeneity and mutual exclusivity of CAF marker expression patterns may be associated with unique functions in different types of malignancy. In breast cancer, CAFs positive for both FAP and podoplanin are immunosuppressive through a nitric oxide (NO)-dependent mechanism [22]. In prostate cancer, CAFs expressing high levels of CD90 play a pivotal role in promoting tumor progression through the upregulation of angiogenic factors, activation of the Hedgehog (Hh) signal, and decreased androgen receptor signaling [23]. In pancreatic ductal adenocarcinoma (PDAC), a specific subpopulation of CAFs was identified that is distinct from myofibroblastic CAFs strongly expressing α-SMA. These inflammatory CAFs express pro-inflammatory cytokines such as interleukin-6 (IL-6) and IL-11, thereby activating the Janus kinase (JAK)/signal transducer and activator of transcription (STAT) signaling pathway [24].

Therefore, this novel review article focuses on critical signal pathways for CAFs to regulate the malignant phenotype, given the crosstalk between tumor cells and CAFs as well as the heterogeneity of CAF population.

## Heterogeneity of CAFs in the tumor-associated stroma

Increasing evidence strongly suggests that CAFs have diverse functions, implying that tumor-promoting CAF and tumor-suppressing CAFs coexist in the tumor stroma [7, 25, 26]. Current cancer immunotherapy strategies primarily target programmed cell death-1 ligand-1 (PD-L1), chimeric antigen receptors, and cytotoxic T lymphocyte-associated antigen 4 [27, 28]; however, the effects of CAFs on tumor immunosuppression remain relatively unexplored. FAP-positive CAF populations drive immunosuppression and promote resistance to anti-PD-L1 immunotherapy [29, 30]. Targeting the C-X-C motif chemokine 12 (CXCL12)–C-X-C chemokine receptor type 4 (CXCR-4) axis with AMD3100 (Plerixafor) reverses FAP-positive CAF-mediated immunosuppression and synergizes with anti-PD-L1 immunotherapy in PDAC [29]. Biffi et al. recently identified IL-1 and transforming growth factor-β (TGF-β) as tumor-secreted cytokines that promote CAF heterogeneity [31]. TGF-β signaling inhibits IL-1 receptor 1 (IL-1R1) expression, antagonizes IL-1α responses, and promotes the differentiation of paraneoplastic fibroblasts into myofibroblastic CAFs. By contrast, an IL-1-induced signaling cascade that activates JAK/STAT promotes the generation of inflammatory CAFs [31]. Therefore, IL-1α signaling is a potential therapeutic target against PDAC cells and inflammatory CAFs in the tumor microenvironment. Elyada et al. used single-cell transcriptomics to examine CAF heterogeneity associated with PDAC and identified a novel CAF population characterized by high expression levels of major histocompatibility complex class II [32]. These antigen-presenting CAFs can present antigens to CD4-positive T lymphocytes.

Costa et al. used multicolor flow cytometry to identify four subtypes of CAFs (CAF-S1, 2, 3, and 4) associated with breast cancer [30] that show differential expression of CAF marker molecules such as α-SMA, caveolin-1 (Cav-1), FAP, and PDGFRβ. CAF subset maps confirmed that CAF-S2 is enriched in luminal-type breast cancer, whereas CAF-S1 and CAF-S4 accumulate in triple-negative breast cancer. The CAF-S1 type attracts CD4 + CD25+ T lymphocytes and retains them through OX40L, PD-L2, and JAM2. CAF-S1 cells promote the inhibition of T effector proliferation by regulatory T cells (Treg). Mechanistically, Costa et al. identified dipeptidyl peptidase 4, a FAP-dimerization counterpart, as a key player in CAF-S1-mediated Treg activation [30, 33]. Givel et al. identified four subpopulations of CAFs in mesenchymal-type high-grade serous ovarian cancer (HGSOC) according to α-SMA, CD29 (integrin β-1), FAP, and FSP1 expression levels [34]. CAF-S2 and CAF-S3 are defined as non-activated CAFs because they have low levels of α-SMA, whereas CAF-S1 and CAF-S4 are considered activated CAFs with high α-SMA expression levels. FOXP3-positive T lymphocytes are enriched exclusively in the CAF-S1 population, which differs from the CAF-S4 subtype in mesenchymal-type HGSOC. The chemokine CXCL12β is expressed at higher levels in CAF-S1 than in CAF-S4, which is associated with the poor prognosis of HGSOC [34, 35]. The CXCL12β isoform is regulated by microRNA (miR)-141/200a; the miR-200 family members miR-141 and miR-200a are responsible for the downregulation of CXCL12β in CAF-S4, whereas miR141/200a promote the specific accumulation of CACL12β in the CAF-S1 subpopulation, inducing the infiltration of CD4 + CD25+ T lymphocytes [34]. Taken together, these data indicate that the antagonistic effects of CAFs on the malignant phenotype may be related to the existence of subpopulations of CAFs with opposing functions.

Recent PDAC studies challenged the concept of tumor-promoting CAFs based on data showing increased tumor growth and aggressiveness following eradication of α-SMA-expressing CAFs and/or targeting of the desmoplastic response induced by the Hh signaling pathway [36, 37]. PDAC lesions with approximately 80% depletion of α-SMA-positive interstitial myofibroblasts show an activated EMT program associated with increased numbers of cancer stem cells (CSCs) and upregulation of EMT-related transcription factors such as Snail, Slug, and Twist. Clinically, lower CAF numbers are correlated with decreased survival in patients with PDAC [36]. Although sonic hedgehog (Shh) ligand and downstream signaling are induced de novo in preneoplastic lesions linked to pancreatic intraepithelial neoplasia and increase significantly during PDAC progression as the stromal compartment enlarges [38], a Shh-depleted PDAC mouse model showed Slug and Zeb1 upregulation leading to poorly differentiated histology [37]. Mizutani et al. identified Meflin as a functional marker of tumor-retarding CAFs in PDAC [39]. Meflin, a glycosylphosphatidylinositol-anchored protein, is a marker of mesenchymal stem cells (MSCs) and maintains their undifferentiated state [40]. In situ hybridization analysis revealed an inverse correlation between α-SMA and Meflin expression in PDAC-associated CAFs. Kaplan-Meier survival analyses showed that high expression levels of Meflin in surgically resected human PDAC tissues are positively correlated with better prognosis, and Meflin-high PDAC displays a more differentiated pathohistology than the Meflin-low group. This suggests that the phenotype of Meflin-high CAFs is distinct from that of tumor-promoting CAFs with high α-SMA expression levels. The PDAC-associated stroma in Meflin-KO genetically engineered model mice shows straighter and wider collagen structures than those of tumors in Meflin-WT PDAC model mice. Lineage-tracing experiments indicate that Meflin-lineage stromal cells contain α-SMA-positive CAFs, which downregulate Meflin and upregulate α-SMA in response to TGF-β and tumor stiffness. This explains why the stromagenic switch, in which tumor-restricting CAFs with high Meflin expression generate tumor-promoting CAFs, contributes to CAF heterogeneity during tumor progression.

## Crosstalk between tumor cells and CAFs

Increasing evidence suggests that CAFs contribute to collective cell migration and invasion by remodeling the ECM to create tracks for tumor cell migration and/or by expressing different cadherins that enable cells to retain adhesion while controlling front/rear polarization of the leading cells [41–43]. Labernadie et al. showed that CAFs increase the invasive potential of tumor cells through N-cadherin upregulation [42]. Intercellular physical force is transmitted between cancer cells and CAFs by a heterophilic adhesion complex involving E-cadherin at the cancer cell membrane and N-cadherin at the CAF membrane. This heterotypic cancer cell-CAF interaction triggers β-catenin recruitment, α-catenin/vinculin interaction, and actin remodeling, allowing CAFs to exert an intercellular physical force on cancer cells and promote cooperative tumor invasion [42].

CAFs contribute to the ‘education’ of carcinoma cells into an invasive and metastatic phenotype [9, 44]. TGF-β and CXCL12 secreted by CAFs enhances the metastatic potential of breast cancer cells undergoing incomplete EMT. Although developmental cells undergo complete EMT during embryogenesis, which is characterized by the cadherin switch, tumor cells express both epithelial and mesenchymal markers [epithelial/mesenchymal (E/M) hybrid phenotype] concurrently, which is defined as “partial EMT” in the process of invasion and distant metastasis [45, 46]. Indeed, circulating tumor cells that survive in the bloodstream show an E/M hybrid phenotype, become resistant to anoikis, and exit the bloodstream more efficiently [46, 47]. CAFs stimulate the invasion of E/M hybrid-type breast cancer cells, which are associated with epithelial-type cancer cell clusters, leading to collective invasion of both epithelial and E/M hybrid tumor cell clusters [48, 49]. Chen et al. recently reported that the epithelial-to-mesenchymal plasticity of lung cancer cells established from a patient-derived xenograft (PDX) is enhanced in the presence of CAFs under three-dimensional culture [50, 51]. CAFs antagonize the oncogenic transcriptional factor SOX2 to restore the formation of luminal structures and promote invasion. Stromal cell-derived factor 1 promotes EMT and increases the stemness of lung squamous cancer cells (LSCCs). Most LSCCs express E-cadherin, and only a small population is positive for vimentin [50]. This finding suggests that spheroids derived from PDX are heterogeneous. The presence of tumor cells positive for both E-cadherin and vimentin suggests that partial EMT occurs in the original tumor, PDX model, and spheroids [46].

CAFs play an important role in the establishment of the omental tumor microenvironment in ovarian cancer. Omental fibroblasts contribute to the creation of a pre-metastatic niche, and influence tropism for the omentum and the metastatic colonization of ovarian tumor cells [52]. Ovarian cancer-derived lysophosphatidic acid and exosomes promote the differentiation of adipose-derived MSCs into CAFs [52–54], which are characterized by the expression of α-SMA, FAP, FSP1, and PDGFR, by activating TGF-β-related signaling pathways [6–8]. Furthermore, ovarian cancer cells reprogram normal omental fibroblasts into CAFs by upregulating miR-155 and downregulating miR-31 and miR-214 [55]. This action promotes tumor proliferation by increasing the secretion of CCL5. Ovarian cancer-derived TGF-β is involved in stimulating the production of various tumor-promoting factors including IL-6, CXCL12, and VEGF-A in the metastatic tumor microenvironment [56]. Omental dissemination induced by this cascade is driven by overexpression of HOXA9 in ovarian cancer cells. CAF-derived TGF-α promotes the metastatic colonization of ovarian cancer cells via the activation of the Akt, epidermal growth factor receptor (EGFR), and extracellular signal-regulated kinase (ERK)-1/2 signaling pathways [57]. Metastasizing ovarian cancer cells can activate p38α MAPK signaling in omental CAFs, and CAF-derived p38α MAPK-regulated cytokines and chemokines, including IL-6, CCL5, and CXCL10, induce glycogen metabolism in cancer cells via glycolysis, which mediates energy production and promotes the aggressiveness of ovarian cancer cells [58]. Furthermore, the differential expression patterns of monocarboxylate transporters (MCT) in cancer cells and CAFs contribute to metabolic symbiosis, in which CAFs depend on aerobic glycolysis and secrete lactate via MCT4 [9, 59, 60]. This “reverse Warburg effect” enables MCT1-positive CSCs to play a fundamental role in maintaining the hierarchy in tumor cellular society unlike MCT4-positive CAFs [59]. In addition, CAFs tend to exhibit robust activity regarding aerobic glycolysis as well as Atg5/7-dependent selective autophagy because of the loss of Cav-1 expression [9, 61, 62]. Such stromal autophagy generates building blocks from recycled free amino acids, fatty acids, and nucleotides, which can be directly utilized by tumor cells to sustain growth and maintain cellular viability. Therefore, CAFs evolve with ovarian cancer cells in the intraperitoneal metastatic microenvironment and govern the metastatic cascade, including the adhesion, proliferation, invasion, and colonization of metastatic sites [52].

Podoplanin-positive CAFs drive tumor progression in a xenograft model, and podoplanin expression in CAFs predicts a poor outcome in patients with lung adenocarcinoma [63, 64]. However, CAFs positive for podoplanin are more frequent in poorly differentiated adenocarcinoma. Clinical cases characterized by the presence of podoplanin-expressing CAFs display a poor response to EGFR tyrosine kinase inhibitors (EGFR-TKIs) in patients with lung adenocarcinoma harboring constitutively active mutations of *EGFR* [65]. By contrast, knockdown of podoplanin makes CAFs susceptible to EGFR-TKIs [66]. Direct contact between cancer cells and CAFs is necessary for acquired resistance to EGFR-TKIs.

## Significance of EGFR signaling in CAFs

The epidermal growth factor receptor (EGFR) belongs to the ErbB family of receptor tyrosine kinases (RTKs) and exhibits critical functions in the epithelial cell physiology [67]. Ligand-dependent activation of EGFR transduces multiple signaling pathways such as PI3K/Akt and Ras/MAPK pathways [68]. Canonical EGFR signaling is essential for several cellular functions including differentiation, proliferation and survival [67]. Notably, increased EGFR expression is positively correlated with reduced recurrence-free and overall survival periods in several kinds of malignancy [69].

**Fig. 2:**
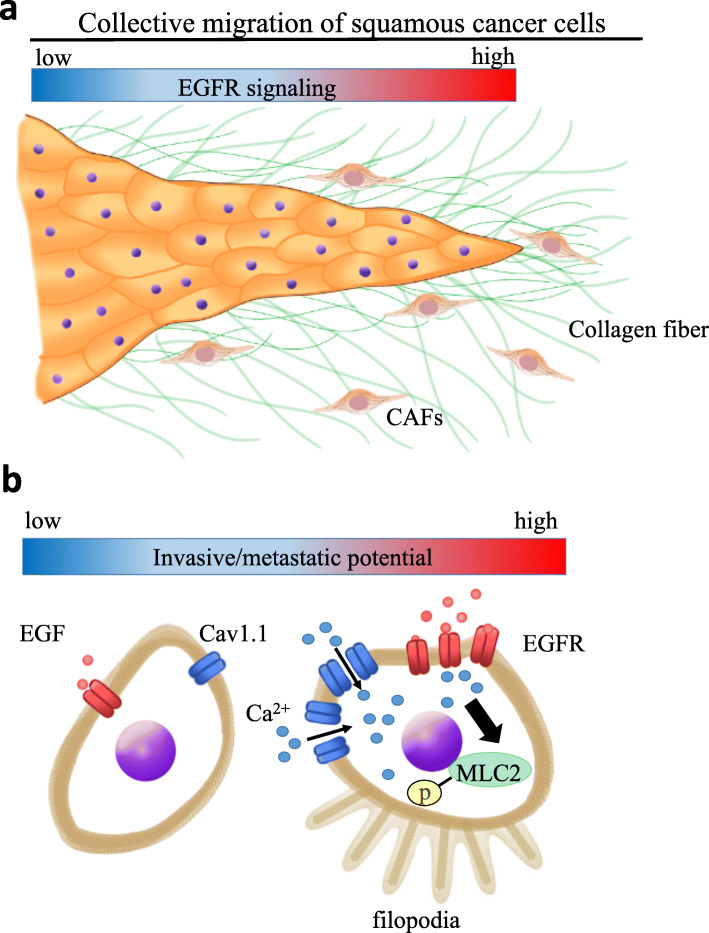
Collective migration of squamous cancer cells (SCCs) is driven by the matrix-dependent mechano-sensitization of EGF signaling. **a** Collective migration of epithelial tumor cells is a tissue-driven process dictated by ECM remodeling, which is orchestrated by CAFs. CAFs lead the way for cancer cells by digging tracks within the ECM that SCCs use to invade [70, 71]. **b** Calcium ions are intracellular second messengers that modulate actomyosin contractility. ECM stiffness and activation of the EGFR signaling pathway increase intracellular calcium concentration mediated by Ca_v_1.1 expression in SCCs [70]. EGF stimulation promotes myosin light chain 2 (MLC2) phosphorylation only when SCCs are exposed to ECM stiffness. Note that ‘p’ indicates phosphorylation

Grasset et al. demonstrated that collective invasion of squamous cancer cells (SCCs) is driven by the matrix-dependent mechano-sensitization of EGF signaling [70] (Fig. 2a). Increasing evidence suggests a connection between mechanotransduction and receptor tyrosine kinase (RTK) signaling pathways. RTKs are activated by dimerization and are involved in integrin-mediated mechanotransduction signaling, which promotes tumor progression [72]. Induction of collagen crosslinking results in stiffness of the ECM, promotes focal adhesion kinase (FAK) expression, increases phosphoinositide 3-kinase activity, and promotes the invasion of oncogene-initiated epithelial cells. By contrast, suppression of integrin signaling inhibits the invasion of a premalignant epithelium into a stiffened, crosslinked stroma. Cell-to-ECM adhesion favors EGFR-dependent cancer proliferation [73]. Because RTKs interact exclusively with active integrins, the composition of the ECM determines the type of RTK/integrin interaction occurring at the cellular membrane. This selectivity may change the intracellular location or conformation of EGFR, thereby changing the accessibility of the receptor intracellular domain to downstream signaling molecules. One of the downstream proteins is FAK, which is targeted to sites of integrin/RTK complex formation and is essential for the transmission of motility signals from EGFR [73, 74]. Furthermore, the *EGFR* gene is amplified, overexpressed, or mutated in SCCs, such as head and neck squamous cell carcinoma (HNSCC) [75, 76]. In the clinical setting, *EGFR* amplification predicts sensitivity to gefitinib in HNSCC [76]. EGFR activation and expression levels are positively correlated with poor prognosis of breast cancer and HNSCC independently from anticancer therapeutics [77]. Grasset et al. identified an association between EGFR activity and stromal stiffness during collective cellular migration [70]. The degree of EGFR signaling is positively correlated with collective cell migration (Fig. 2a). The L-type calcium channel Ca_v_1.1 is a critical regulatory element during the collective invasion of squamous cell carcinoma and acts downstream of ECM stiffness and EGFR signaling both in vitro and in vivo. The L-type calcium channel Ca_v_1.1 is a critical regulator of SCC collective migration in response to stromal stiffness and EGFR signaling activation (Fig. 2b), and calcium channel blockers, which are widely used for the treatment of arrhythmia and hypertension, are promising therapeutic agents against SCC invasion and metastasis. EGFR blockage induces EMT and CAF activation in HNSCC [78], and the calcium channel antagonists verapamil and diltiazem reduce resistance to EGFR-targeting treatments [70]. This is an example of drug repositioning, namely, screening for the anticancer therapeutic effects of conventionally administered medications for non-malignant disorders [59, 62]. Increased rigidity in the tumor stroma favors EGFR activity and results in the calcium-dependent regulation of Cdc42 small GTPase activity in tumor cells. This signaling route is critical for HNSCC cell invasion into the stiffened stroma. Although myosin light chain (MLC) kinase, an important regulator of actomyosin contractility in cancer cells [79], does not play a role in collective SCC migration, Cdc42 finely regulates the actomyosin-dependent remodeling of the ECM by CAFs [70]. PDX models developed in verapamil- or diltiazem-treated mice show reduced levels of phosphorylated MLC2 and a decrease in the number of filopodia, which regulate tumor cell invasion [51, 70] (Fig. 2b).

More recently, Gao et al. have demonstrated that CAFs associated with HGSOC contribute to the formation of heterotypic spheroids in the malignant ascites [80]. Those CAFs-centered spheroids recruits floating ovarian cancer cells, resulting in the formation of ‘metastatic units’ at early stages of transcoelomic metastasis [80, 81]. Mechanistically, floating ovarian cancer cells drive the production and secretion of EGF by CAFs located at the center region of the spheroids. This consequently promotes ITGA5 (integrin α5) expression in tumor cells, which in turn further enhances the tumor-stromal interaction inside the heterotypic spheroids [80]. That is why EGF is expected to the promising therapeutic target to prevent the peritoneal dissemination of HGSOC. Indeed, it has been shown that a neutralizing anti-EGF antibody can suppress the formation of spheroid in ascites mediated by the attenuated expression of ITGA5 in floating ovarian cancer cells, leading to the prolonged survival period [80].

## Significance of canonical Wnt signaling in CAFs

Signal activation due to the Wnt family of secreted glycolipoproteins is one of the crucial machineries underlying the cellular polarity, proliferation, and cell fate determination during the embryonic development and tissue homeostasis [82]. In the absence of Wnt ligand, cytoplasmic β-catenin is degraded constantly by Axin complex, which is composed of Axin, APC, casein kinase 1 (CK1), and glycogen synthase kinase 3 (GSK3) [82]. CD44, c-Myc, Axin2, and Cyclin D1 are the typical target molecules regulated by the nuclear translocation of β-catenin. Interestingly, reactive oxygen species (ROS) activates canonical β-catenin-dependent Wnt signal pathway [83, 84], which is responsible for up-regulation of c-Myc at the invasive front enriched in cancer stem-like cells [85, 86].

Ferrari et al. recently reported that Dickkopf (DKK)-3, which activates the canonical Wnt signaling pathway, is highly expressed in CAFs in breast, colon, and ovarian cancer stroma [87]. DKK1 expression is downregulated in fibroblasts in pachydermoperiostosis (PDP), a rare chronic inflammatory disease characterized by unique skin and bone phenotypes associated with loss-of-function mutation of the *HPGD* gene, thereby increasing the proliferation capacity of PDP-associated fibroblasts [88, 89]. In contrast to DKK1, DKK2, and DKK4, which suppress Wnt/β-catenin signal transduction, DKK3 does not interact with LDL-receptor-related protein (LRP) 5/6 and therefore cannot fulfill the bona fide antagonistic role of the DKK family in the canonical Wnt signaling pathway [90, 91]. Instead, DKK3 decreases the stability of Kremen, a Wnt negative regulator, resulting in increased LRP6 membrane localization, which in turn stabilizes both β-catenin and Yes-associated protein (YAP)/transcriptional coactivator with PDZ-binding motif (TAZ) levels [87, 91]. Of note, heat-shock factor 1 (HSF1) interacts with the enhancer and promoter regions of the *Dkk3* locus, and contributes to the upregulation of DKK3 in CAFs. HSF1 promotes tumor growth by inducing cytokines such as CXCL12 and TGF-β [92]. Although β-catenin-mediated Wnt signaling is dispensable for the function of CAFs in remodeling the ECM and promoting tumor cell proliferation and invasion, DKK3-driven YAP activation is necessary to induce a tumor-promoting phenotype [87]. Absence of DKK3 in DKK3-null normal fibroblasts and CAFs is associated with decreased YAP/TAZ and β-catenin activity. By contrast, depletion of DKK3 leads to the concomitant upregulation of Kremen, LRP6 inactivation, and destabilization of both β-catenin and YAP/TAZ in CAFs. This DKK3-mediated localization and stabilization of YAP/TAZ in the nucleus is independent from the Hippo pathway, in which phosphorylated YAP (Ser127) plays a central role [93]. Thus, DKK3 is expected to mediate the crosstalk between Wnt/β-catenin signaling and YAP/TAZ.

Periostin, which is abundantly produced and secreted by CAFs in HNSCC, promotes the CSC phenotype via the canonical Wnt/β-catenin signaling pathway [94]. Periostin is highly expressed in the tumor stroma compared with cancer cells and promotes tumor progression and metastasis in HNSCC [94, 95]. Yu et al. showed that periostin secreted by CAFs is a potential ligand for protein tyrosine kinase 7 (PTK7), which is frequently upregulated in HNSCC tissues and is correlated with Wnt/β-catenin pathway activation and poor clinical outcome in HNSCC patients [94]. CAF-derived periostin is highly likely to bind to PTK7 on the cancer cell membrane and transduces signals to disheveled protein through the cell surface receptor LRP6; this induces the phosphorylation of GSK-3β and the hypophosphorylation of β-catenin, which causes β-catenin to translocate into the nucleus, suggesting that the periostin-PTK7 axis activates the canonical Wnt signaling pathway [94]. The periostin-PTK7 axis promotes tumorigenesis, lung metastasis, and chemoresistance mediated by β-catenin expression in HNSCC. Thus, treatment with a PTK7 neutralizing antibody increases the therapeutic efficacy of erlotinib, a small-molecule TKI effective for the treatment of metastatic and/or recurrent HNSCC, by downregulating β-catenin [94, 96].

## Significance of Hippo signaling in CAFs

Hippo signal pathway is an evolutionary well-conserved regulator of organ size, which is first discovered in *Drosophila*. Central to this signaling is a kinase cascade leading from the tumor suppressor Hippo (Mst1/ Mst2 in mammals) to the oncogenic Yki (YAP/TAZ in mammals), which is a transcriptional coactivator of target genes involved in cell proliferation and survival [93]. The major target transcription factors regulated by YAP and TAZ are the four proteins of the TEA-domain-containing (TEAD) family (TEAD1-TEAD4). While Mst1/2 is downregulated in several kinds of carcinomas, TAZ has been reported to be upregulated in invasive breast cancer [97].

The Hippo pathway is activated by stromal stiffness in solid tumor tissues, and a growing body of evidence suggests that the transcriptional factor YAP is activated in CAFs [13, 98, 99]. YAP/TAZ is activated in response to mechanical stress and perturbation of the actin cytoskeleton [100]. YAP/TAZ activation by mechanical stimuli in cells is influenced by Rho-GTPase, Rho-associated protein kinase (ROCK), and the integrity of the actomyosin cytoskeleton in a manner largely independent from the large tumor suppressor kinase [99]. Pathohistological analysis of normal murine mammary tissues and PyMT-driven breast tumors shows nuclear accumulation of YAP in the stroma of both adenoma and carcinoma lesions [98]. YAP activation in the stroma is further enhanced in the peripheral tumor regions of advanced carcinomas such as breast cancer and squamous cell carcinoma. YAP controls the expression of several cytoskeletal regulators including ANLN, connective tissue growth factor (CTGF), and diaphanous homolog 3 (DIAPH3), and then regulates the expression levels of MYL9/myosin light chain (MLC)-2. Matrix stiffening promotes YAP activation, thereby establishing a positive-feedback loop that helps maintain the CAF phenotype [98]. Increased interstitial fluid pressure (IFP) blocks the delivery of therapeutic agents, whereas reduced tumor IFP improves the uptake of chemotherapeutic drugs [101, 102]. Therefore, lowering tumor interstitial hypertension, which acts as a barrier against tumor transvascular transport, has been proposed as a general strategy to increase tumor uptake as well as the therapeutic effects of anticancer drugs. Blocking the tyrosine kinase PDGFRβ increases the susceptibility to conventional chemotherapy in a xenograft model of anaplastic thyroid carcinoma [101].

**Fig. 3:**
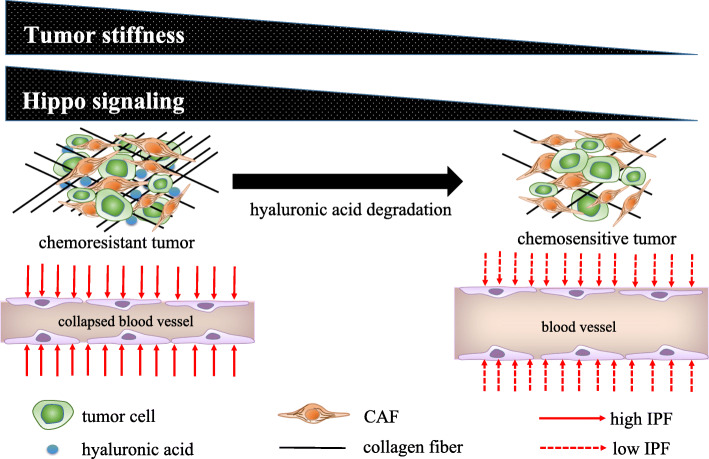
Degradation of hyaluronic acid renders chemoresistant tumors sensitive to anticancer drugs by attenuating the collapse of blood vessels. The stroma associated with pancreatic ductal adenocarcinoma (PDAC) shows a robust and complex desmoplasia, with notable hyaluronic acid (HA) content and the collapse of blood vessels in response to high interstitial fluid pressure (IFP). Stromal stiffness is positively correlated with the Hippo pathway. PEGPH20 contributes to the enzymic degradation of HA and decreases the degree of stromal stiffness [102]. The attenuated IFP broadens the lumen of tumor vessels, thereby increasing the efficacy of drug delivery. A clinical trial of PEGPH20 in advanced solid tumors is ongoing [107]

YAP activation serves as an independent prognostic marker for the overall survival of PDAC patients and its association with liver metastasis [103]. Hyaluronic acid (HA) is the major determinant of elevated IFP in PDAC. In addition, the presence of α-SMA-positive myofibroblasts increases the number of fibrotic foci and promotes contraction of the interstitial space [8]. The increased number of blood vessels together with increased hydraulic conductivity or the relative ease with which fluid moves across the vessel wall is responsible for an irregular and increased influx of fluid into the tumor stroma. Increased IFP is frequently reported in solid tumors such as breast carcinoma, glioblastoma, and malignant melanoma [104–106]. Enzymatic degradation of HA results in the rapid reduction of IFP and the appearance of widely patent functioning vessels in the tumor microenvironment [102]. Removing these barriers permits high concentrations of chemotherapy agents to reach PDAC tissues, which improves survival and reveals an unappreciated sensitivity of the disease to conventional cytotoxic agents (Fig. 3). In the clinical setting, the combination of gemcitabine and PEGPH20 has attracted attention for the treatment of stage IV PDAC because of its effect on HA degradation in the tumor stroma [102, 107].

## Significance of TGF-β signaling in CAFs

TGF-β signal pathway contributes to the maintenance of tissue homeostasis and prevention of incipient malignancy by regulating not only cellular adhesion, differentiation, proliferation and survival, but also the microenvironment [108]. Injured epithelial tissue is gradually repaired by the formation of granulation tissues composed of α-SMA-positive myofibroblasts, macrophages, platelets, newly formed blood vessels and ECM [9] (Fig. 1). Pathological forms of TGF-β signal pathway drive tumor growth and invasive phenotype, evasion of immune surveillance, and distant metastasis including cancer cell dissemination [108]. As with the wound-healing process, tumor-derived TGF-β is likely to recruit other stromal cell types characterized by CAFs and osteoclasts, which are enriched at the invasive front and at the bone metastatic disease, respectively.

The dependence of myofibroblastic CAFs on autocrine TGF-β signaling remained unclear until a study demonstrated that the establishment of self-sustaining CXCL12 and TGF-β autocrine signaling pathways results in the formation of tumor-promoting CAFs during breast cancer progression [109]. The two autocrine signaling pathways triggered by CXCL12 and TGF-β may promote CAF differentiation associated with increased α-SMA expression levels [109]. TGF-β and CXCL12 secreted by cancer cells upregulate CXCR4 and stabilize the Smad-dependent TGF-β pathway.

Loss of Cav-1 in the tumor stroma activates TGF-β signaling in CAFs [9, 110]. Activation of the TGF-β ligand by proteolytic cleavage promotes its interaction with specific receptors. TGF-β binds to TGF-β receptor type II, and promotes the formation of a hetero-oligomeric complex with TGF-β receptor type I, resulting in the activation of the TGF-β receptor kinase. The TGF-β receptor in turn phosphorylates serine/threonine residues in downstream target effectors such as Smad proteins. The activated TGF-β receptor complex initiates several downstream cascades, including the canonical Smad2/3 signaling pathway and non-canonical pathways, such as TGF-β-activated kinase-mediated p38- or JNK-signaling [111, 112]. Activation of TGF-β signaling leads to EMT in cancer cells, which express high levels of matrix metalloproteinase (MMP) [113, 114]. Discoidin domain receptor 2 upregulates MMP2 and MT1-MMP in a manner dependent on the ERK2/SNAIL1 axis in hepatocellular carcinoma [114, 115].

**Fig. 4:**
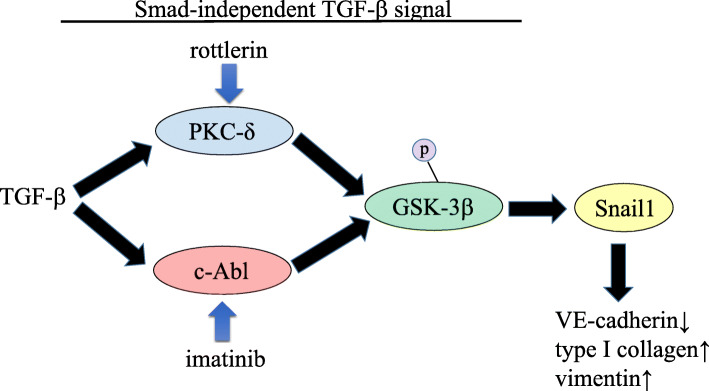
A non-Smad TGF-β signaling pathway is involved in tissue fibrosis by promoting endothelial-mesenchymal transition (EndoMT). Following ligand-initiated activation of the Smad-independent TGF-β pathway, GSK-3β is phosphorylated by c-Abl and PKC-δ non-receptor kinases. Phosphorylation of GSK-3β at serine 9 (Ser9) causes its inhibition, which allows Snail1 to enter the nucleus. Nuclear accumulation of Snail1 leads to acquisition of the myofibroblast phenotype characterized by stimulation of α-SMA and type I collagen instead of VE-cadherin. The specific inhibition of c-Abl activity with imatinib allows GSK-3β to phosphorylate Snail1, which targets it for proteasomal degradation, thereby abolishing the acquisition of the myofibroblastic phenotype and the fibrotic response. Rottlerin and imatinib inhibit PKC-δ and c-Abl, respectively, which is why both agents abrogate EndoMT [116]. Note that ‘p’ indicates phosphorylation. Black arrows indicate induction or promotion, whereas blue arrows indicate inhibition

Ligand-initiated activation of the Smad-independent TGF-β pathway triggers GSK-3β phosphorylation by c-Abl and PKC-δ non-receptor kinases. Phosphorylation of GSK-3β at serine 9 (Ser9) causes its inhibition, which in turn allows Snail1 to enter the nucleus. Nuclear accumulation of Snail1 leads to acquisition of the myofibroblastic phenotype with stimulation of α-SMA and type I collagen instead of VE-cadherin. Inhibition of c-Abl activity with imatinib allows GSK-3β to phosphorylate Snail1, which targets it for proteasomal degradation and effectively abolishes the acquisition of the myofibroblastic phenotype and the fibrotic response. Rottlerin and imatinib abrogate EndoMT by inhibiting PKC-δ and c-Abl, respectively [116] (Fig. 4).

Mammary fibroblasts in which Cav-1 is depleted show CAF phenotypes, such as the conversion of fibroblasts to myofibroblasts and enhanced TGF-β signaling [117]. Cav-1 directly inhibits TGF-β signaling. Mechanistically, Cav-1 interacts with the TGF-β receptor type 1, inducing its degradation, and suppresses TGF-β-dependent Smad2 phosphorylation and nuclear translocation [118]. Activation of the TGF-β signaling pathway is sufficient to downregulate Cav-1 expression [119], thereby forming a positive-feedback loop involving Cav-1 expression levels and TGF-β signaling activity. Significant downregulation of stromal Cav-1 is responsible for the metabolic reprogramming of CAFs, which is characterized by the induction of aerobic glycolysis (also referred to as “reverse Warburg effect”) and autophagy in the tumor-associated stroma. This results in the stromal production of energy-rich metabolites including L-lactate, pyruvate, and ketone bodies, as well as chemical building blocks such as amino acids (glutamine), nucleotides, and fatty acids [59, 120]. These recycled nutrients are then transferred to adjacent epithelial tumor cells, thereby fueling cancer progression in a paracrine fashion. Because activation of TGF-β signaling attenuates mitochondrial metabolism, and enhances aerobic glycolysis and autophagy (especially mitophagy, in which old dysfunctional mitochondria undergo degradation) [62, 110], CAFs that secrete TGF-β ligands in an autocrine manner can proliferate independently of angiogenesis. Cancer cell-induced ROS promote the loss of stromal Cav-1 in fibroblasts via autophagy and activate hypoxia-inducible factor α (HIF1-α) under ROS-induced pseudohypoxic conditions [9, 59, 121]. This phenomenon is termed metabolic symbiosis [9, 59]. Enhanced expression levels of MCT4 and BNIP3 in CAFs are responsible for the activation of aerobic glycolysis via metabolic symbiosis and mitophagy, respectively [9, 59, 110].

## Significance of JAK/STAT signaling in CAFs

JAKs are non-receptor tyrosine kinases mediating signal transduction which is involved in cellular proliferation and survival. The seven mammalian STAT family contain the tyrosine residue near the C-terminus which is phosphorylated by JAK family in the presence of growth factors, interleukins and interferons (IFN) [122]. This phosphorylation allows STATs to form the dimer via the interaction with a conserved SH2 domain. Remarkably, there are several cytokines with distinct, and sometimes opposing, functions are likely to activate the same STAT protein [123, 124]. For a typical instance, IL-6, a pro-inflammatory cytokine which utilizes gp130, promotes the activation of STAT3. In contrast, IL-10, which is a potent anti-inflammatory cytokine, does not utilize gp130 but promotes STAT3 phosphorylation.

Actomyosin contractility plays a key role in tumor cell migration, affecting both the tumor cells themselves and the remodeling of the ECM by tumor fibroblasts to permit cell migration [71]. CAFs remodel the ECM using contractile forces and proteolytic activity, thereby generating tracks for the migration of tumor cells as collective strands led by a fibroblast [43]. Force-mediated matrix remodeling largely depends on integrins α3 and α5, as well as Rho-mediated regulation of MLC activity in fibroblasts. However, these factors are not required in cancer cells. Instead, tumor cells depend on Cdc42 and myotonic dystrophy kinase-related Cdc42-binding protein kinase (MRCK)-mediated regulation of MLC to follow the tracks generated by fibroblasts in the ECM. Force-mediated matrix remodeling by CAFs depends on actomyosin contractility modulated by the ROCK signaling pathway [43, 125]. Rab21-positive vesicles preferentially localize to the areas of cell contraction, and both integrin α5 and Rab21 are required for MLC phosphorylation [125]. Rab21 delivers integrin α5 to the cellular membrane, where it signals to the contractile machinery. At least three Rab proteins, including 5a, 11b, and 21 subtypes, are needed in CAFs for their ability to promote SCC invasion. Depletion of these Rab proteins does not affect the ability of SCC cells to invade the ECM previously remodeled by CAFs. This can be attributed to the fact that the ‘following’ SCC cells do not remodel the matrix, and matrix remodeling pathways are therefore dispensable in SCC cells. Cytokine signaling through GP130-IL6ST and JAK1 stimulates actomyosin-mediated contractility in cancer cells and in the tumor-associated stroma [71]. GP130-IL6ST signaling to JAK1 drives actomyosin-mediated contractility in CAFs and promotes matrix remodeling. JAK1 signaling regulates actomyosin contractility by regulating the level of phosphorylated-MLC2 in both melanoma cells and CAFs, the latter of which lead SCC invasion. Pro-inflammatory cytokines, such as IL-6 and LIF, are aberrantly expressed by CAFs in the tumor microenvironment and induce chemoresistance as well as EMT [126, 127]. The axis involving the cytokine oncostatin M (OSM) acts through GP130-IL6ST, JAK1, and ROCK to drive actomyosin contractility and matrix remodeling by CAFs for the collective invasion of SCCs [71]. OSM induces fibrotic changes in the lungs and liver, and promotes EMT and the myofibroblastic phenotype via the JAK/STAT axis, thereby predisposing to cancer development [128, 129]. JAK/STAT signaling may involve a Rho guanine nucleotide exchange factor, ARHGEF1, to activate RhoA to the GTP-bound state as in vascular smooth muscle cells. This is supported by data showing that basal RhoA activity in CAFs is sensitive to inhibition of JAK, and OSM activates RhoA in a JAK-dependent manner [71, 130]. Unlike melanoma cells, in which GP130-IL6ST/JAK1-ROCK signaling is required for cancer cell migration, this signaling pathway is not necessary in tumor cells, whereas it is required in CAFs for ECM remodeling leading to the collective invasion of SCCs [71]. Therapeutic agents, including blocking antibodies against cytokines, such as IL-6, or small molecule inhibitors of JAK kinase or STAT activity, could be useful agents to block invasion and metastasis in malignant diseases. IL-6 receptor blockage inhibits lung metastasis of breast cancer cells by suppressing IL-6-induced JAK/STAT signaling [131]. Furthermore, an anti-IL-6 neutralizing antibody named siltuximab inhibits non-small cell lung cancer progression [132].

In an analysis of epigenetic alterations, Albrengues et al. demonstrated that aberrant DNA methylation maintains the phenotype of tumor-promoting CAFs via the JAK/STAT cascade [133]. JAK1/STAT3 signaling is constitutively activated in CAFs, partly because STAT3 acetylation induced by CBP/p300 leads to the epigenetic-dependent loss of expression of Src homology phosphatase-1 (SHP-1), which is a negative regulator of JAK/STAT signaling. SHP-1, also known as tyrosine-protein phosphatase non-receptor type 6, dephosphorylates several tyrosine kinases including JAK1 [134]. Silencing of SHP-1 by DNA methyltransferase 1-mediated promoter hypermethylation leads to the sustained constitutive phosphorylation of JAK1 kinase and the STAT3 transcription factor, which maintain the contractile and pro-invasive properties of CAFs [133]. Pharmacological inhibition with both 5-azacytidine and ruxolitinib results in the long-term abrogation of JAK1/STAT3 phosphorylation and rescues the expression of SHP-1, thereby inhibiting the tumor-promoting invasive phenotype of CAFs. Given that genetic mutations are rare in CAFs, further investigations are warranted to identify epigenetic abnormalities in the cancer-associated stroma [9, 135, 136].

## Conclusion

CAFs contribute to the formation and maintenance of the tumor microenvironment in cooperation with tumor cells by activating several signaling cascades including the EGFR, JAK/STAT, TGF-β, and Wnt pathways. In addition, stromal stiffness leads to drug resistance and poor prognosis of cancer patients. Given that α-SMA-positive activated fibroblasts form a senescence-associated secretory phenotype loop in response to treatment with HDAC inhibitors [137, 138], re-education of the tumor stroma could be a promising therapeutic strategy. Treatment with chemotherapy and/or radiotherapy eradicates responsive diseases. However, survival of CAFs is associated with minimal residual disease. The surviving CAFs acquire innate and adaptive resistance to therapy, which is accompanied by stromal inflammation and increased ECM accumulation, leading to iatrogenic tumor stiffness and the development of chemoresistant tumors [9]. Hirata et al. indicated that CAFs associated with *BRAF*-mutant malignant melanoma are activated in response to PLX4720, a selective BRAF inhibitor. PLX4720 paradoxically activates ERK/MAPK signaling in residual disease, promotes collagen production and matrix remodeling, and promotes MLC phosphorylation [139]. This iatrogenic activation of CAFs is responsible for the FAK-dependent persistent survival of melanoma cells. The ability of melanoma-associated fibroblasts to confer PLX4720 tolerance largely depends on both FAK and integrin β1 in melanoma cells. Furthermore, stiffness of the fibronectin-rich stroma is sufficient to abrogate the effects of BRAF inhibition. PDX models indicate that dual inhibition of BRAF and FAK inhibits ERK/MAPK re-activation in the tumor stroma, which facilitates the efficient therapeutic control of *BRAF*-mutant melanoma [51, 139]. As such, the tumor microenvironment mainly composed of CAFs determines the dynamic phenotype and plasticity of cancer cells in cooperation with intrinsic genetic/epigenetic alterations [140]. The degree of cancer cell differentiation may be largely controlled by the “stromagenic switch”, which results in CAF heterogeneity. In addition, α-SMA-negative and PDGFRβ-positive CAF subpopulations contribute to the malignant potential of tumor cells by interacting with integrin α11 [141, 142]. Of note, α-SMA-negative inflammatory CAFs secrete high levels of pro-inflammatory cytokines such as IL-6, IL-11, and LIF, and activate the JAK1/STAT3 cascade [24]. In verity, several molecular machineries underlying invasive/metastatic phenotype and therapy-resistance driven by CAFs have been uncovered. Surprisingly enough, there exist tumor-restricting CAF populations which have been shown to inhibit tumor growth and progression [143, 144]. Accumulating evidence demonstrates that activation of Hh signal pathway in CAFs suppresses the growth of tumors mediated by bone morphogenetic protein (BMP) signaling in cancer cells, which strongly suggests the presence of CAF populations with tumor-suppressive functions. Taken together, the existence of several potential CAF markers suggests that further investigation is warranted to identify the pathophysiological functions of these molecules.

## Data Availability

The datasets used and/or analyzed during the current study are available from the corresponding author on reasonable request.

## Abbreviations

α-SMA: α-smooth muscle actin
CAFs: Cancer-associated fibroblasts
Cav-1: Caveolin-1
CSCs: Cancer stem cells
CXCL12: C-X-C motif chemokine 12
CXCR4: C-X-C chemokine receptor type 4
DKK: Dickkopf
ECM: Extracellular matrix
EGFR: Epithelial growth factor receptor
EMT: Epithelial-mesenchymal transition
EndoMT: Endothelial-mesenchymal transition
ERK: Extracellular signal-regulated kinase
FAK: Focal adhesion kinase
FAP: Fibroblast activation protein
FSP1: Fibroblast-specific protein 1
GSK-3β: Glycogen synthase kinase 3β
HA: Hyaluronic acid
HGSOC: High-grade serous ovarian cancer
Hh: Hedgehog
HNSCC: Head and neck squamous cell carcinoma
HSF1: Heat-shock factor 1
IFP: Interstitial fluid pressure
IL: Interleukin
JAK: Janus kinase
LIF: Leukemia inhibitory factor
LRP: LDL-receptor-related protein
MCT: Monocarboxylate transporter
miR: microRNA
MLC: Myosin light chain
MMP: Matrix metalloproteinase
MSCs: Mesenchymal stem cells
OSM: Oncostatin M
PDAC: Pancreatic ductal adenocarcinoma
PDGFR: Platelet-derived growth factor receptor
PD-L1: Programmed cell death-1 ligand-1
PDX: Patient-derived xenograft
PTK7: Protein tyrosine kinase 7
ROCK: Rho-associated protein kinase
ROS: Reactive oxygen species
RTK: Receptor tyrosine kinase
SCC: Squamous cancer cell
Shh: Sonic hedgehog
SHP-1: Src homology phosphatase-1
STAT: Signal transducer and activator of transcription
TAZ: Transcriptional coactivator with PDZ-binding motif
TKI: Tyrosine kinase inhibitor
TGF-β: Transforming growth factor-β
Treg: Regulatory T cell
YAP: Yes-associated protein
